# Localization of Ion Concentration Gradients for Logic Operation

**DOI:** 10.3389/fchem.2019.00419

**Published:** 2019-06-06

**Authors:** Nikolay V. Ryzhkov, Pavel Nesterov, Natalia A. Mamchik, Stanislav O. Yurchenko, Ekaterina V. Skorb

**Affiliations:** ^1^Laboratory of Solution Chemistry of Advanced Materials and Technologies, ITMO University, Saint Petersburg, Russia; ^2^Terahertz Technology Lab, Bauman Moscow State Technical University, Moscow, Russia

**Keywords:** interface, polyelectrolyte multilayers, pH-gradient, logic gates, iontronics

## Abstract

Adjustment of the environmental acidity is a powerful method for fine-tuning the outcome of many chemical processes. Numerous strategies have been developed for the modification of pH in bulk as well as locally. Electrochemical and photochemical processes provide a powerful approach for on-demand generation of ion concentration gradients locally at solid-liquid interfaces. Spatially organized in individual way electrodes provide a particular pattern of proton distribution in solution. It opens perspectives to iontronics which is a bioinspired approach to signaling, information processing, and storing by spatial and temporal distribution of ions. We prove here that soft layers allow to control of ion mobility over the surface as well as processes of self-organization are closely related to change in entropy. In this work, we summarize the achievements and discuss perspectives of ion gradients in solution for information processing.

## Introduction

Nobel laureate Herbert Kroemer stated that “the interface is the device” in reference to heterogeneous semiconductor structures. But this idea also inspired the development of interfacial science beyond the physics of heterostructures. Interfaces play a significant role in many physical and chemical processes. There is a wide variety of procedures for surface treatment and the modification of functional interfaces. Decher et al. (1992) described alternately exposing of charged substrates to positively and negatively charged macromolecules in order to obtain functional multilayered coatings. Layer-by-layer (LbL) assembled coatings and capsules have found various applications due to the versatility of multilayer formation technique and a variety of charged compounds which may be incorporated into it (Decher et al., 1998; Ryzhkov et al., 2019a). Polyelectrolyte multilayers are traditional components of biomaterial surface coatings (Zhukova et al., 2017), membranes for separation (Rmaile et al., 2003), as well as cargoes for drug encapsulation and delivery (Nikitina et al., 2018).

Polyelectrolyte multilayers are also considered to be an appropriate model mimicking the structure and properties of biological membranes (Zhu and Szostak, 2009). A lipid bilayer supported by a polyelectrolyte cushion provides a platform for modeling and investigating many cellular processes. Nowadays, transport processes in polyelectrolyte layers has garnered much attention. Due to their selective ionic permeability (Tanaka and Sackmann, 2005), one may perform dynamic polarization across the membrane. This process simulates neuron polarization during nerve conduction, thereby modeling information processing in living systems.

Development of a reliable model infallibly mimicking biological way of information processing is still a challenge. A lot of efforts are put to mimic biological way of computation by artificial matter. The simplest example of information operations is switching functions following Boolean logic—logic gates. Logic gates use binary inputs and produce a single binary output. By now, several systems based on polyelectrolytes that perform information processing according to Boolean logic and using ionic signals (particularly protons) have been developed. Motornov et al. (2008) designed an enzyme-based hybrid system of pH-responsive nanoparticles assembling and disassembling following AND/OR Boolean logic. Motornov et al. (2009) also developed a pH-responsive Pickering emulsion coupled with specific enzymatic reactions performing AND and OR logic. Han et al. (2009) developed microchip polyelectrolyte diodes representing AND, OR, and NAND logic based on ion transportation through the polyelectrolyte interface. Thus, applicability of iontronic devices were demonstrated.

Reversible conformational changes of polymer brushes atop an electrode modulated by pH and influencing charge transport behavior are wifely exploited for designing biomimetic iontronic calculating devices. A more detailed mechanism is described as follows. For example, sucrose in the presence of both invertase (Input A) and glucose oxidase (Input B) results in a decrease in pH, transforming into gluconic acid (AB). The described system performs AND Boolean logic. OR logic can be designed using an ethyl butyrate-glucose mixture. Acidification of the medium (A+B) in this case can be achieved either by the oxidation of ethyl butyrate to butyric acid by esterase (Input A) or by the oxidation of glucose to gluconic acid by glucose oxidase (Input B). In brief, enzymatic inputs run a cascade of reactions leading to a pH shift to acidic values. The electrode surface with grafted shrunk poly-4-vinyl pyrrolidone (P4VP) at neutral pH is not electrochemically active because of the blocking effect of the polymer film. Output “1” of the logic operations yielded a pH drop to acidic conditions, resulting in the protonation and swelling of the P4VP polymer allowing penetration of a soluble redox probe to the conducting support. Thus, one may perform amperometry or impedance spectroscopy study for output detection. Wang et al. (2009) designed the system described above. Different groups employed a large variety of enzymes and electrode coatings for realizing a similar approach to designing biochemical logic gates. Privman et al. (2008), Katz and Minko (2015), and Poghossian et al. (2015) developed enzyme-based biocomputing systems coupled with pH-responsive membranes and electrodes. As a result, they obtained bioelectronic devices switchable by logically processed biomolecular signals. Reversible pH-responsive on-off behavior performing Boolean logic was suggested for designing novel multi switchable electrochemical biosensors based on electrodes covered by polymer network (Liu et al., 2012) or polymer brushes (Li et al., 2014) and electrodes made of inorganic-polymer composites (Wang et al., 2015).

Significant progress in DNA and molecular logic operations was made in recent years. However, various issues remain unresolved. For example, information transfer through live-machine interfaces: living matter conducts electricity mostly using ions, while machines conduct electricity mostly using electrons (Yang and Suo, 2018). It's of high importance nowadays because technologies at the interface between natural and artificial plays a central role in science. We suggest here a strategy for performing iontronic logic operations at interfaces in solutions and present it's modeling by the electrochemical and photochemical system and how they can be related to Shannon's entropy. Having an array of electrodes and applying potential bias according to some program, one may realize the spatial distribution of acidic and basic areas in water solutions close to an electrode in a unique pattern with micrometer resolution. It's key to iontronic information processing. Designating current density higher than some value as logic input “1” and pH lower than some cut-off as logic output “1,” we may design different logic gates varying the geometry of input electrodes, the position of response point and cut-off values. We present here the simplest AND logic gate performed by a couple of microelectrodes and discuss assembling of individual logic gates in concatenated logic cascades and complex branching networks by multielectrode arrays.

Having an array of electrodes and applying potential, oxidation, and reduction processes may be localized at particular electrodes and hence spatially separated. Protons propagating from parental electrode serve as information transmitters. Moreover, we have demonstrated that polyelectrolyte modification of electrode surface may lead to amplification and better localization of ion fluxes and hence signal amplification and can be related to self-organization *vs*. Shannon's entropy. All this discussion is also extrapolated to polyelectrolyte modified photoelectrodes under irradiation.

## Enhancing Electrochemically Produced Ionic Signals by Polyelectrolyte Assemblies

Electrochemistry provides one with a powerful approach for the on-demand local generation of ionic signals. For example, proton fluxes may be produced by electrochemical hydroquinone oxidation (Fomina et al., 2016). Due to their proton-coupled electron transfer, low redox potential, and relative chemical stability, quinones are widely used as the electroactive species for the controlled generation/consumption of protons (Dochter et al., 2015; Garnier et al., 2015). Naturally occurring hydroquinone compounds play a significant role in electron/proton transfer of many biological processes (Jeyanthi et al., 2016). Thus, the quinone/hydroquinone transition serves as an essential electrochemical model for the development of biomimetic systems. Its changing molecular structure allows for its electrochemical properties to be tuned (Peduto et al., 2017). But characteristics of an electrode reaction are highly affected by the microstructure of the electrode surface, since the fact that electrochemical systems under investigation are heterogeneous and electrode reaction is related to electron transfer through the electrode-electrolyte interface.

**Figure 1:**
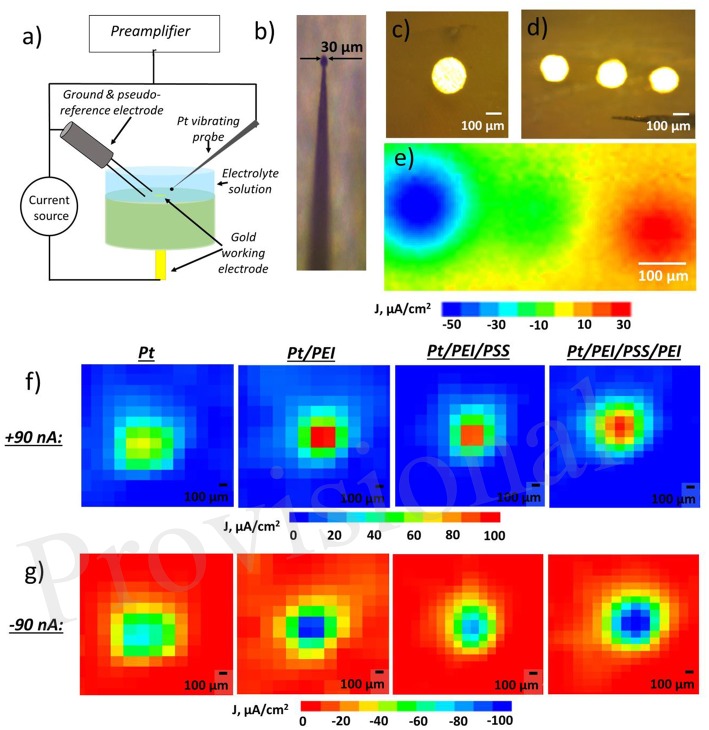
**(a)** Schematic of scanning vibrating electrode technique (SVET) for determining an ionic current that can be used together with scanning ion selective electrode (SIET). **(b)** Pt-Ir vibrating probe for SVET, **(c)** top view of gold electrode embedded in epoxy resin, **(d)** top view of working electrodes (WE) embedded in epoxy resin **(e)** ion currents mapped by SVET in solution over three WEs, left electrode is negatively polarized (−90 nA), right one—positively (+90 nA), red demonstrates areas of positive ionic current in solution related to anodic activity and blue—areas of negative ionic current in solution and cathodic activity on the surface, **(f,g)** ionic current over one WE under either positively [in **(f)**] or negatively [in **(g)**] on pristine Pt or Pt covered with different polyelectrolyte multilayers—Pt/ polyethylenimine (Pt/PEI), Pt/PEI/ poly(sodium 4-styrenesulfonate) (Pt/PEI/PSS), and Pt/PEI/PSS/PEI—number of layers affects drastically on the ionic current.

Electrode surface influences diffusion of electrochemical reactants and products as well as Faradaic process. In case of hydroquinone oxidation, horizontal and vertical proton propagation is supposed to be a three-dimensional pH wave. It originates from the ion source and weakens as it moves away. Fick's laws describe diffusion and postulate that ionic flux goes from regions of higher concentration to areas with a lower one. Furthermore, the magnitude of the driving force determining ion movement is proportional to the concentration gradient. Ion fluxes and the concentration of a particular ion close to the surface of the electrodes were investigated using Scanning Vibrating Electrode Technique (SVET) and Scanning Ion-Selective Electrode Technique (SIET), unique tools for the characterization of local ionic currents in solution and ion concentration gradient measurement, respectively (Souto et al., 2010). SVET allows the electric field in a solution to be measured for the visualization of anodic and cathodic areas on surfaces with nA precision and μm spatial resolution (Figure 1a). During SVET-analysis, a vibrating Pt-probe (Figure 1b) scans the surface, estimating its electrical potential in amplitude points of its vibration and then recalculates it in ionic currents. SIET is based on potentiometric principles. A glass capillary microelectrode with the ion-selective membrane in the tip scans the surface, measuring the concentration of a particular ion.

We have studied the effect of polyelectrolyte assembly on top of an electrode on the propagation of electrochemically generated protons. The system under investigation is a working electrode (WE) gold or platinum wire embedded in an epoxy resin so that its round section was brought into contact with a solution (Figure 1c). System may be also extended to several electrodes (Figures 1d,e). Electrochemical reaction is coupled with proton release. The pH-wave propagation is driven by a concentration gradient. Further, the surface of the noble metal electrode (WE) was subsequently modified with nanometer-thin layers of the polycation polyethyleneimine (PEI) and the polyanion poly(sodium 4-styrenesulfonate) (PSS). PEI is considered to be a proton sponge that stores electrochemically generated protons while the PSS layer serves as a cation exchange layer. The electrode was first covered by branched PEI to provide secure anchoring to the surface and to act as a positively charged terminating layer. Deposition of PSS was then carried out via electrostatic interaction with the underlying layer. Although polymer multilayer assembly leads to no change in redox processes at electrode/electrolyte interface, both the anodic and cathodic activity of the Pt electrode measured by SVET is higher for one that is polyelectrolyte coated than for a bare one (Figures 1f,g). It is worth noting that a terminating PEI layer resulted in more pronounced anodic/cathodic activity. However, if this polyelectrolyte membrane is thick enough, ion flux from the surface is suppressed (Ryzhkov et al., 2019b).

Since LbL polyelectrolyte assemblies contain many uncompensated charges, ions cannot freely pass through the membrane. Although it is still passive transport down their concentration gradient, ion movement pathways are more complex and cannot be explained by simple diffusion. We consider polyelectrolyte LbL assemblies as a convenient tool for controlling the transmission of the ion signal. Strong and weak polyelectrolyte assemblies, including charged biopolymers and hydrogels, can regulate charge carrier generation, the diffusion of ions at interfaces, lifetime and storage (Ryzhkov et al., 2019a). There is still no general theory precisely predicting electrode response to polyelectrolyte modification. Until now, multicomponent coatings formed by multilayers of different polyelectrolyte compositions (strong–strong, strong–weak, weak–weak) have been analyzed as nanolayers for corrosion protection. The mechanism of multilayer protective action is based on pH buffering polybasic and polyacid complexes (Andreeva et al., 2010; Skorb and Andreeva, 2015). It has also been demonstrated that polyelectrolyte layers can be used as an efficient pH-buffering protective layer for pH-sensitive soft materials (Skorb and Andreeva, 2013). It is expected that by combining polyelectrolytes of different molecular weights, strengths, and with different specific functional moieties, one can flexibly tune spatial and temporal distribution of ionic fluxes through the membrane and perform independent handling by cations and anions. That fact opens up prospects for developing futuristic biomimetic information processing using ions as signal carriers.

## Discussion of Soft Matter Assemblies for Controlling Ion Fluxes

LbL deposition of polyelectrolyte multilayers is a universal approach to designing interfaces with unique properties. Its impact is not limited by enhancement of ion fluxes described above. Different effects may be achieved by polyelectrolytes of different nature. Recent progress in science provides an understanding of polyelectrolyte complexation principles. Despite the apparent simplicity of the assembly procedure, the processes occurring in the multilayers are incredibly complex. Polyelectrolyte layers in multilayered structures are not perfectly stratified, and polymer chains of polycations and polyanions are significantly interpenetrated (Schönhoff, 2003). Much attention is drawn to the dynamics and internal structure of polyelectrolyte multilayers and studying of internal diffusion (Fares and Schlenoff, 2017; Selin et al., 2017). Various parameters such as ionic strength (Steitz et al., 2000), charge density (Steitz et al., 2001), pH, and temperature (Karg et al., 2008) influence the internal structure of polyelectrolyte film. Several models of diffusion in ultrathin polyelectrolyte films were suggested (Klitzing and Möhwald, 1996; Farhat and Schlenoff, 2001). Three different modes of interaction of polyelectrolyte multilayers and small ionic species were observed—permeability, non-permeability, and ions accumulation. It was also found that the permeability of polyelectrolyte membranes depends mostly on film composition rather than its thickness (Hoshi et al., 2003). The outermost layer of polyelectrolyte LbL assembly carry out excess non-compensated charge and plays a crucial role in permeability properties blocking penetration of similarly charged small species (Rmaile et al., 2003). Fu et al. (2017) demonstrated that pairs of weak polyelectrolytes tend to transport small molecules or ions more, whereas multilayers assembled from strong polyelectrolytes are less permeable. Kelly et al. (2018) demonstrated that ion flux through the membrane is significantly affected by the stoichiometry of the polyelectrolyte multilayer. An excess of some component, polycation, or polyanion, changes diffusion and permeability of ions through the polyelectrolyte multilayer. Ion transportation through polyelectrolyte multilayers can be described similarly to solid matter permeability. Thus, the action of surrounding polyelectrolyte chains affects diffusion through polyelectrolyte assemblies significantly and diffusion is thermoactivated (Spruijt et al., 2008). Internal interfaces predetermine the properties of polyelectrolyte multilayers, and one should therefore keep in mind the composition and structure of the multilayer, the internal layer chemistry, and interactions between components when designing functional polyelectrolyte multilayers (Brezhneva et al., 2019). The means of changing the permeability of polymer layers mentioned above have already found extensive use in the development of semipermeable separating membranes and electrochemical sensors with improved selectivity, sensitivity, and response time.

Thus, polyelectrolyte multilayers are a powerful instrument for the regulation of ion-fluxes. By varying membrane composition, enhanced ion transport, accumulation, and delayed release can be realized.

## pH-wave Propagation as a Basis for Iontronics

Precise control over electrochemically generated ion fluxes open perspectives for flexible and reliable approach for transition from machine way of information processing (via electrons) to biological one (via ions) and developing technologies at the interface between natural and artificial (wearable and implantable devices, for example).

**Figure 2:**
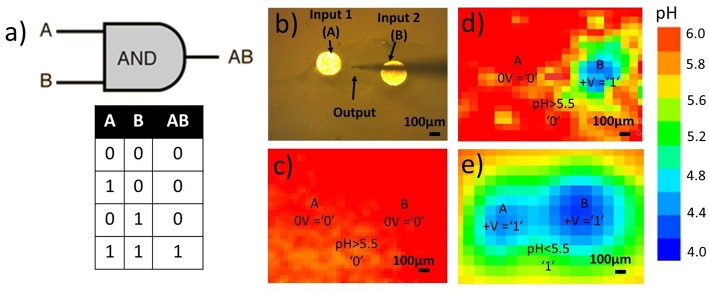
**(a)** Basic AND logic gate and corresponding truth table, **(b)** system of two WEs embedded in epoxy holder, **(c–e)** 2D pH-maps via SIET of two input electrodes in hydroquinone solution during polarization of electrodes in different regimes and interpretation in terms of logic gates, positive polarization +V is determined as input “1,” no polarization 0V as input “0,” pH>5.0 is determined as output “0” and pH <5.0 as output “1,” **(c)** no potential applied, both inputs are “0,” as a result, no pH drop – output “0,” **(d)** positive potential is applied to the right electrode “1,” left one is not polarized “0,” resulting pH drop localized on right electrode and doesn't propagate to output area “0” **(e)** positive potential applied to both electrodes “1,” resulting proton wave reaches output area giving output “1”.

Our interest is focused on information transfer in aqueous solutions and the prospective for communication with living matter. Here, we demonstrate a proof of concept of basic logic operations that use ions as input and/or output signals which allows unequivocal output reading. The system under investigation is presented by an array of gold or platinum electrodes particularly embedded in an epoxy resin and immersed in the electrolyte solution. The simplest model systems containing two electrodes are shown in Figure 2.

By applying positive and negative potentials to the electrodes one is able to carry out a pH coupled redox process (e.g., hydroquinone oxidation). Herewith, oxidation and reduction processes may be spatially separated. As a result, the distribution of acidic and basic areas in a solution adjusted to an electrode surface may be realized particularly. Anodic and cathodic activity are localized directly at the electrodes while the resulting pH gradient is more spatially blurred. The desired localization of the proton wave may be achieved via electrode functionalization by polymer assembly. The electrodes may be designated as inputs and the acidity of the space between the electrodes as output in terms of logic gates. Simple AND logic operations (Figure 2a) may be performed. The main processor of any computing device is basically a bunch of interconnected logic gates, thus performing these simple logic operations is an important step toward biomimetic iontronic calculations. The open-circuit potential applied to the input electrode (Figure 2b) is designated as input “0” and hydroquinone oxidation potential (0.70 vs. SHE) as input “1.” The acidity of the solution between the electrodes is read as an output signal. A pH lower than some threshold, for example, 5.0, is designated as output “1” with anything lower being “0.” Two “0” inputs provide “0” output (Figure 2c). If only one of the electrodes is polarized, the resulting pH wave does not reach the output area, and pH > 5.0. Therefore, the output signal is “0” (Figure 2d). Otherwise, if both input electrodes are polarized, generated protons propagate to the output area, making the pH there significantly acidic, <5.0, giving signal “1” in output area (Figure 2e).

In general, the model system described above may be extended to several dozen electrodes. As such, some electrodes may be assigned as inputs while others are for the reading of the electrochemical output. We started with three microelectrodes (Figure 1d) and, by SVET, demonstrated that the independent polarization of electrodes might be performed, while no effect of the bipolar electrode was observed in the studied potential window (Figure 1e).

What we plan to do next is to cover the output electrode with pH sensitive film, grafted P4VP or P2VP brushes for example (Pennakalathil et al., 2010; Ghostine and Schlenoff, 2011). This thin polymer layer in its non-protonated state is collapsed and acts as an insulator, inhibiting direct electron transfer from the electrode to the electrochemically active specimen in solution and vice versa. If only one input electrode is active, the resulting proton wave does not reach the output electrode. The polymer layer still blocks the electrode surface and no current is observed during polarization. When both inputs are “1” (applied potential of hydroquinone oxidation), then the resulting proton wave reaches the output electrode, making the surrounding media acidic enough to protonate the blocking polymer layer atop the output electrode. As a result, polymer conformation changes from collapsed to swelled, allowing penetration of hydroquinone to the electrode. Thus, an anodic current of hydroquinone oxidation may be registered at the output electrode. A Faradaic current registered at the output electrode above a certain threshold is assigned as “1” and lower as “0.” Thus, switching of output electrode activity may be performed according to Boolean logic. It is worth noting that in this case some autocatalysis may be shown, and the acidification of the area close to the output electrode leads to electrochemical generation of more protons. Thus, signal amplification and signal transmission from one location to another realizing specific pathways through the electrode network may be performed.

Our future research direction will be focused on different geometries of input electrode array, varying applied potentials and passed currents, and regimes of application (constant current, pulses, etc.). Another direction is the development of novel approaches for output electrode modification for ensuring disambiguation of output response reading.

## Self-Organization vs. Shannon's Entropy

Fundamental concept of information theory is Shannon's entropy. Entropy in this case is a measure of unpredictability of the state, or equivalently, of its average information content. We suggest here description of described above approach to designing of iontronic devices in terms of Shannon's entropy.

**Figure 3:**
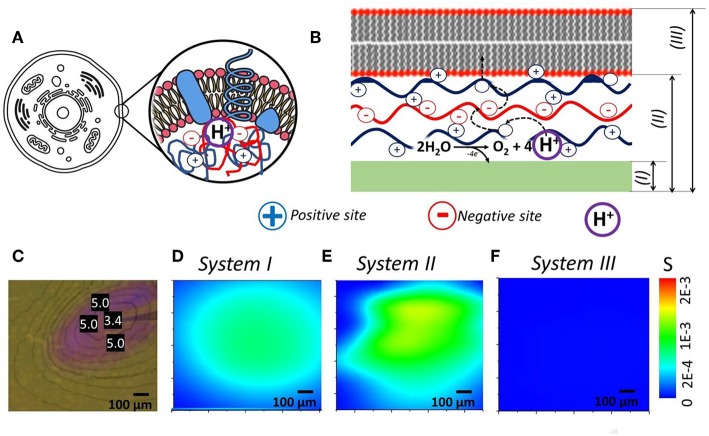
**(A,B)** Idea of cell prototype system that consists of polyelectrolyte assembly or coacervates, reactions inside that are protected from environment by lipid bilayers. **(C)** System with local gradient of pH initiated by light (measured pH values are shown in units in photo). **(D–F)** Recalculated into Shannon's entropy SIET pH maps of—**(D)** pristine WE, **(E)** WE covered with polyelectrolytes, and **(F)** WE covered with polyelectrolytes and lipid bilayer—processes of self-organization are closely related to change in entropy of the system.

Idea here is a correlation of soft matter components (polyelectrolytes and lipid layers) of living cell and our biomimetic model (Figures 3A,B). We took photochemical system (Figure 3C) (Maltanava et al., 2017) shown previously as the analogy to electrochemically induced proton gradients in aqueous electrolytes and collect SIET pH maps for pristine working electrode (WE) (System I), WE covered with polyelectrolyte multilayers (System II) and WE covered with polyelectrolyte multilayers and lipid layer on top (System III).

Processes of self-organization are known to be closely related to change in entropy of the system (Haken, 2006). Typically, this occurs with spatial change in thermodynamic (as temperature, density, and pressure) as well as chemical parameters and, in particular, ion composition of the system. In our case, the changes of temperature, density, and pressure are negligible, while the local electrochemical influence affects directly the ion-distribution in the solution. Note that the spatial pH-redistribution occurs self-consistently, being accompanied by local electric potential redistribution during the free-energy minimization of the system. In result, the self-organization and the redistribution of pH-fields are directly associated with each other and, thus, the change of entropy can be illustrated using the fields of pH.

According to its definition, the Shannon's entropy is (Haken, 2006).

(1) $$\begin{array}{r} S=-\sum_{i}p_{i}\log_{2}p_{i} \end{array}$$

where 0 < *p*_*i*_ < 1 is the probability to measure some observable value *i*. In a case of a lot of observable independent values (e.g., set of *p*-values at different spatial points), corresponding summation over them should be performed in the right hand side of Equation (1).

The particular physical sense of the probability *p* depends on the system's nature and plays an important role for interpretation of the results. For instance, *p* can be related to the probability of some molecular dipole orientation or electric charge in case of electric systems, an electron spin orientation or magnetic polarization in magnetic systems, a particular state of photons in optical systems, or concentration of chemicals in reacting systems (Haken, 2006). In the same manner, this approach could be naturally expanded to solutions, to consider *p* in sense of probability that an observed ion in the solution is an H^+^ or OH^−^ ion (whose concentrations are related with each other). In other words, we may use the parts of H^+^ and OH^−^ ions as the probabilities p_H_ and p_OH_, to calculate corresponding contribution to the Shannon's entropy associated with the pH in a given spatial area of measurement. One should note that, generally speaking, the pH distribution provides the same information as the field of electric potential, since they are consistently related with each other in the solution.

The probability to observe H^+^ cations in a solution during a measurement is simply related to the cation concentration in the system and pH of the solution as P_H_ = C_H_/C_0_ = 10^−pH^, where C_0_ = 1M is a normalizing concentration. For OH^−^ anions, we have p_OH_ = C_OH_/C_O_ = 10^pH−14^, since p_H_ p_OH_ = 10^−14^. From here, by substitution of this expression for p into Equation (1), we readily obtain the pH-related part of the Shannon's entropy as

(2) $$\begin{array}{l} \text{S}={\text{log}}_{2}\text{​}10\;(\text{pH }10^{-\text{pH}}+\text{pOH }10^{-\text{pOH}})\\ \;={\text{log}}_{2}\text{​}10\;(\text{pH }10^{-\text{pH}}+(14-\text{pH})\;10^{\text{ pH}-14}) \end{array}$$

Equation (2) determines the contribution to Sannon's entropy, associated with pH measured in a small volume (which we consider as a subsystem of a large system herein the pH-measurement is performed). At pH = 7, Equation (2) exhibits a local minimum. Note that Equation (2) is symmetric relatively pH = 7 and, at pH < 6.8 (pH > 7.2), the first (second) term in the parenthesizes becomes negligible.

The total entropy for given discrete spatial distribution of pH (distribution of the system states) can be calculated with summation over the *S*-values in all spatial points of the system. This situation with discrete distribution of pH field is typical for experiments we performed, since the size of a “cell” (small open volume of the solution) wherein pH is measured is determined by the electrode size.

The approach based on Equation (2) is convenient for analysis of self-organization phenomena (related to change in pH-distributions) and their interpretation in terms of entropy fields. This can be illustrated using results of our measurements of pH fields in different systems. For instance, taking spatial distribution of cations determined experimentally in cases of bare electrode, as well as for electrode covered by polyelectrolyte multilayer (PEI/PSS)_3_ and lipid bilayer, we obtained the spatial distributions of entropy *S*(*x, y*) represented in Figures 3D–F. Interestingly, huge difference is observed for obtained the spatial distributions of entropy that can be associated with various soft matter components for controlling ion fluxes.

## Concluding Remarks and Outlook

In this perspective, we highlight information processing and signaling by spatial and temporal distribution of ions. A model electrochemical and photochemical system creating local ion-fluxes were demonstrated.

Electrodes spatially organized in a particular manner allows propagation of pH waves to be triggered and spatial distribution of H^+^ according to a particular pattern to be realized. Proton diffusion from two sources is reported to model the AND logic gate. We are currently studying how the system geometry influences the pattern of proton concentration and developing a simulation that predicts pH pattern depending on the working electrode geometry and vice versa, namely suggesting electrode geometry depending on desired pH pattern.

The LbL assembly of polyelectrolyte multilayers is suggested as an instrument to control horizontal and vertical ion propagation with ability to correlate it with the spatial distributions of entropy. The experiments we have described are only a small sample of the full range of polyelectrolyte materials that can be assembled on top of electrodes and tested for ion conduction ability.

## Experimental Section

Three-electrode electrochemical cells (working electrode, Pt counter electrode and Ag/AgCl reference electrode) were utilized as model electrochemical and photoelectrochemical systems. Working electrode was presented by gold or platinum wire (0.2 mm in diameter, 2–3 cm length) embedded in epoxy resin so that circular cross-section of wire exposed to outside media on flat surface of obtained holder. Anodized TiO_2_ (1.5 cm^2^) under low intensity light-emitted diode (365 nm) irradiation focused in spot (~0.25 cm^2^) was utilized as working photoelectrode. The anodic and cathodic activity of electrode under polarization in water solution as well as photoactivity of illuminated TiO_2_ was studied by SVET and generated pH gradients by SIET. To perform the SVET and SIET measurements, a system from Applicable Electronics (USA) modulated by an ASET program (Sciencewares, USA) was used. As a vibrating probe for SVET experiments, an insulated Pt-Ir microprobe (Microprobe Inc., USA) with a platinum black spherical tip 30 μm in diameter was used. The probe was made to vibrate both parallel and perpendicular to the specimen surface at a height of 150 μm. The amplitude of vibration was 30 μm, while the probe vibrated at frequencies of 136 Hz (perpendicular to surface) and 225 Hz (parallel to surface). Only the perpendicular component was used in the treatment and presentation of the data. The environmental pH measurements by SIET were carried out using glass-capillary microelectrodes filled with Hydrogen Ionophore Cocktail I (Sigma) based liquid pH-selective membrane and KCl + KH_2_PO_4_ internal solution. Ag/AgCl/KCl (sat) was used as the external reference electrode. The pH-selective microelectrodes were calibrated using commercially available pH buffers and demonstrated a linear Nernstian response−55 to−58 mV/pH—in a pH range from 3 to 8. The local activity of H^+^ was detected 25 μm above the surface. Instrumentation allows to measure voltage with nV precision level, and measure extremely low ion concentration gradients. Step motors allow to study electrochemical activity of material with micrometer spatial resolution and high-resolution maps were obtained. Electrochemical systems were studied in 60 mM hydroquinone solution in 150 mM KNO_3_, photoelectrochemical one without addition of hydroquinone.

The deposition of the polyelectrolyte multylayers onto the surface of working electrode was performed using the classical Layer-by-Layer technique. Two mg/ml each branched polyethylenimine (PEI, Mw 70 kDa, 30% water solution purchased from Alfa Aesar) and polystyrene sulfonate (PSS, Mw 500 kDa purchased from Polysciences Inc.,) were dissolved in 0.5 M aqueous NaCl to make polycation and polyanion solutions respectively. Each layer took 20 min to be deposited after which it was rinsed with excess distilled water and then steam-dried. On top of polyelectrolyte modified TiO_2_ lipid bilayer was also deposited from 10 mg/ml dispersion of Lecisoy 400 vesicles for 1 h. Further electrochemical characterization of modified electrodes and photoelectrodes were performed as described above.

SVET data presented as obtained, SIET data recalculated according to previous calibration. Mapping for each experimental condition were reproduced at least three times, one of typical maps is presented.

## Author Contributions

NR and ES contributed conception and design of the study. SY contributes discussion and writing of Shannon's entropy vs. H^+^ concentration gradient part. NR, NM, and PN performed experimental work and treatment of the data. NR wrote the first draft of the manuscript. ES coordinated the study and helped draft the manuscript.

### Conflict of Interest Statement

The authors declare that the research was conducted in the absence of any commercial or financial relationships that could be construed as a potential conflict of interest.
